# Prevalence of elevated microsatellite alterations at selected tetranucleotide repeats in pancreatic ductal adenocarcinoma

**DOI:** 10.1371/journal.pone.0208557

**Published:** 2018-12-07

**Authors:** Taiki Mori, Yasushi Hamaya, Takahiro Uotani, Mihoko Yamade, Moriya Iwaizumi, Takahisa Furuta, Hiroaki Miyajima, Satoshi Osawa, Ken Sugimoto

**Affiliations:** 1 First Department of Medicine, Hamamatsu University School of Medicine, Hamamatsu, Japan; 2 Laboratory Medicine, Hamamatsu University School of Medicine, Hamamatsu, Japan; 3 Center for Clinical Research, Hamamatsu University School of Medicine, Hamamatsu, Japan; 4 Department of Endoscopic and Photodynamic Medicine, Hamamatsu, Japan; National Cancer Center, JAPAN

## Abstract

Pancreatic ductal adenocarcinoma (PDAC) prognosis remains poor even after complete resection owing to no valuable biomarkers for recurrence and chemosensitivity. Tumors not expressing MSH3 show elevated microsatellite alterations at selected tetranucleotide repeats (EMAST). EMAST reportedly occurs in several tumors. In colorectal cancer (CRC), EMAST was reportedly correlated with 5-fluorouracil (5-FU) sensitivity. However, EMAST prevalence in PDAC and its significance as a prognostic biomarker are unknown. This study aimed to investigate EMAST prevalence in PDAC and the associations between EMAST and pathological factors, EMAST and prognosis, and EMAST and MSH3 expression via immunohistochemistry (IHC). We assessed 40 PDAC patients undergoing surgery. Genomic DNA was extracted from tumors and normal tissues. EMAST and microsatellite instability-high (MSI-H) were analyzed using five polymorphic tetranucleotide markers and five mononucleotide markers, respectively. Tumor sections were stained for MSH3, and staining intensity was evaluated via the Histoscore (H-score). Eighteen of 40 (45%) PDAC patients were EMAST-positive; however, none were MSI-H-positive. Clinicopathological characteristics including overall survival (OS) and recurrence-free survival (RFS) were not significantly different between EMAST-positive and EMAST-negative patients (P = 0.45, 0.98 respectively). IHC was performed to evaluate MSH3 protein expression levels for the PDAC tissue specimens. H-scores of EMAST-positive patients ranged from 0 to 300 (median, 40) and those of EMAST-negative patients ranged from 0 to 300 (median, 170). MSH3 protein was not significantly downregulated in EMAST-positive patients (P = 0.07). This study is a preliminary study and the number of cases investigated was small, and thus, study of a larger cohort will reveal the clinical implication of EMAST.

## Introduction

Pancreatic ductal adenocarcinoma (PDAC) is the fourth leading cause of cancer-related mortality in the United States, with a 5-year survival rate of 8% [1]. Surgical resection is the only curative treatment. PDAC is difficult to diagnose owing to obscure and nonspecific symptoms. Notwithstanding advancements in diagnostic modalities, such as computed tomography, magnetic resonance imaging, positron emission tomography, endoscopic ultrasonography, methods have yet to be standardized for primary screening of the asymptomatic phase [2, 3]. Consequently, fewer than 20% of patients are eligible for curative surgical resection because a majority of PDAC patients have advanced disease at the time of diagnosis [4].

In addition to surgical treatment, patients with resectable PDAC were administered adjuvant or neoadjuvant chemotherapy, and patients with unresectable PDAC were also administered chemotherapy. The lack of valuable biomarkers for cancer treatment and prognosis remains a significant concern [5].

A microsatellite is a region of repetitive DNA containing certain DNA motifs (1–6 bp or more)[6]. Microsatellite instability (MSI) is a form of genomic instability resulting from alterations in the length (increased or decreased) of microsatellite repeats. Loss of DNA mismatch repair (MMR) functions causes the accumulation of point mutations and insertion/deletion loops of one or a few base pairs [7]. Thus, loss of MMR function causes MSI [8].

Several proteins such as hMLH1, hMSH2, hMSH3, and hMSH6, are involved in MMR. Among them, the complex of hMutSα and hMutSβ comprises heterodimers of MMR proteins hMSH2-hMSH6 and hMSH2-hMSH3, respectively [9, 10]. The hMutSα complex recognizes most single-base mismatches [9, 11]. However, hMutSβ identifies larger insertion/deletion loops (IDLs) [9, 11, 12].

Elevated microsatellite alterations at selected tetranucleotide repeats (EMAST) are identified owing to frameshift mutations in tetranucleotide repeats of DNA sequences [13]. MutSβ has a potent affinity for recognizing more than two unpaired nucleotides [11, 12]. Therefore, MSH3 deficiency in human cells increases the instability at loci containing tetranucleotide repeats [14–16]. One of the causes of the deficiency and downregulation of MSH3 is considered to be a frameshift mutation in the A (8) tract of exon 7 of *MSH3* in microsatellite instability-high (MSI-H) tumors [17]. EMAST occurs in multiple solid organ tumors such as colorectal cancer (CRC), lung cancer, ovarian cancer, prostate cancer, renal cancer, and endometrial cancer [18]. Clinicopathological factors are reportedly correlated with EMAST status in CRC patients [19].

Only 1–2% of PDAC patients presented MSI-H [20–22]. Compared to MSI-H, the prevalence of EMAST has not been reported in detail in the past. PDAC is characterized by hypovascular tumors and dense desmoplastic stroma [23]. Furthermore, the hypoxic state or inflammation in tissues decreases MSH3 function [24]. Therefore, some PDAC patients are expected to be EMAST-positive.

The association between EMAST and 5-fluorouracil (5-FU) chemosensitivity remains controversial, and previous studies have reported a correlation between EMAST and 5-fluorouracil (5-FU) sensitivity in CRC [13, 25].

This study aimed to investigate EMAST prevalence in PDAC and the association between EMAST and PDAC prognosis and between EMAST in PDAC and MSH3 expression via immunohistochemistry (IHC).

## Materials and methods

We retrospectively assessed 40 patients who underwent surgical treatment for PDAC at the Hamamatsu University School of Medicine, from 2005 to 2014. This study was approved by the institutional review board of Hamamatsu University School of Medicine. Since we used pre-existing pathological specimens, the institutional review boards waived the need for informed consent from the patients or relatives. Clinicopathological data of patients were used in this study. All patients underwent a radical surgical procedure. Tumor location, patient age, sex, tumor site, administration of chemotherapy, tumor stage, tumor size, and histopathological grade were obtained from the medical records system. Formalin-fixed, paraffin-embedded blocks were prepared from surgical resection samples, and serial sections were prepared for hematoxylin and eosin (H&E) staining. Dissected specimens were deparaffinized in a microfuge tube with xylene, and DNA was purified with ethanol and QIAmp DNA Investigator Kit (Qiagen, Valencia, CA, USA) in accordance with a previous method [16]. EMAST status and MSI-H were determined on the basis of five polymorphic tetranucleotide markers (MYCL1, D9S242, D8S321, D20S82, and D20S85) and five mononucleotide markers (BAT-25, BAT-26, NR-21, NR-22, and NR-25) [16]. Genomic DNA was extracted from tumors and normal tissues, and each of them was amplified via polymerase chain reaction (PCR) with specific primers for each mononucleotide and tetranucleotide marker, using AmpliTaq Gold (Thermo Fisher Scientific, MA, USA) in accordance with the manufacturer’s protocol. PCR was performed using fluorescent-labeled primers. S1 Table enlists the sequence of all primers. The Cycling conditions were as follows: initial denaturation at 95°C for 15 min; 40 cycles at 94°C for 1 min, 55°C for 1 min, and 72°C for 30 s; final extension at 72°C for 10 min. Fluorescently labeled fragments generated via PCR were analyzed using an Applied Biosystems 3130xl Genetic Analyzer with the GeneMapper. We analyzed the PCR products to identify frameshift mutations at mononucleotide and tetranucleotide repeats for each track. When aberrant peak +/- multiples of 4 nucleotides were observed in the electrophoretograms from the tumor compared to control cases, the marker was considered positive for frameshift mutation-induced instability. Tumors with frameshift mutations in at least two markers, compared to control cases, were defined as MSI-H tumors or EMAST-positive tumors, and all others were defined as MSI-L/MSS or non-EMAST tumors.

IHC was performed as described previously [26]. Briefly, tumor tissue sections were immunostained for MSH3 [EPR4334 (2); ab111107; Rabbit monoclonal; 1:500] (Abcam, Cambridge, MA, USA) and detected via streptavidin-biotin-horseradish peroxidase complex formation.

Staining intensity was evaluated using the Histoscore (H-score) [27]. H-score is an assessment method for both staining intensity (graded as: 0, non-staining; 1, weak; 2, median; or 3, strong using adjacent normal mucosa as the median) and the percentage of positive cells. The range of possible scores is 0–300. The expression levels of each component were evaluated.

The statistical software package SPSS 24.0 (SPSS, Inc., Chicago, IL, USA) was used for statistical analysis of the data. Mann‑Whitney's U test was performed to analyze MSH3 expression levels in EMAST-positive tumors versus non-EMAST tumors. Correlations between EMAST status and clinicopathological characteristics were analyzed via Fisher's exact test or Log-Rank test. Correlations between EMAST status and overall survival (OS) or recurrence-free survival (RFS) were analyzed via the Log Rank test. A P‑value less than 0.05 was considered statistically significant.

## Results

We included 40 PDAC patients from the Hamamatsu University School of Medicine and obtained clinicopathological data and tissues for genetic analysis. Table 1 enlists all patients and tumor profiles.

**Table 1:** Clinicopathological characteristic of the pancreatic ductal adenocarcinoma patients.

	All	EMAST	Non-EMAST	
	(n = 40)	(n = 18)	(n = 22)	P value
Age(years),median(range)	71 (45–87)	68 (46–85)	70 (45–87)	0.630
Sex				1.00
Female	23	10	13	
Male	17	8	9	
Tumor site				0.447
Head	30	14	16	
Body	5	3	2	
Tail	5	1	4	
Chemotherapy				0.709
Adjuvant	27	11	16	
Neoadjuvant	1	1	0	
None	12	6	6	
Primary tumor				0.343
T1a	3	2	1	
T1b	0	0	0	
T1c	6	4	2	
T2	16	7	9	
T3	14	4	10	
T4	1	1	0	
Regional lymph Nodes				0.764
N0	14	7	7	
N1	19	9	10	
N2	7	2	5	
Distant metastasis				1.00
M0	37	17	20	
M1	3	1	2	
AJCC prognostic Groups				
(8^th^ edition)				0.591
IA	6	4	2	
IB	3	2	1	
IIA	3	0	3	
IIB	18	8	10	
III	7	3	4	
IV	3	1	2	
Tumor size (mm)				0.399
Median(range)	33 (5–70)	37 (16–70)	30 (5–65)	
Differentiation				0.538
Well	7	2	5	
Moderately	19	10	9	
Poorly	14	6	8	

Among the 40 patients assessed for EMAST, 18 patients (45%) were EMAST-positive, defined as 2 or more tetranucleotide markers showing frameshift mutation [16] (Table 2).

**Table 2:** Status of elevated microsatellite alterations at selected tetranucleotide repeats (EMAST) status in pancreatic ductal adenocarcinoma patients.

Number of marker-positive	Number of patients
0	8
1	14
2	12
3	4
4	2
5	0

We compared EMAST-positive patients with non-EMAST patients, as shown in Table 1. No significant differences were observed between EMAST and non-EMAST patients with regards to sex, tumor location, tumor size, differentiation, or stage (Table 1).

Twenty-two patients (55%) experienced PDAC recurrence. Ten of 22 patients were EMAST-positive, and the others were EMAST-negative. The site of recurrence was local (8 EMAST-positive patients, 3 non-EMAST patients), liver (4 EMAST-positive patients, 2 non-EMAST patients), lymph node (2 EMAST-positive patients, 1 non-EMAST patient), lung (1 EMAST-positive patient), bone metastasis (1 non-EMAST patient), and peritoneal carcinomatosis (1 non-EMAST patient). No significant difference was observed in recurrence status between EMAST-positive and non-EMAST patients.

**Fig 1:**
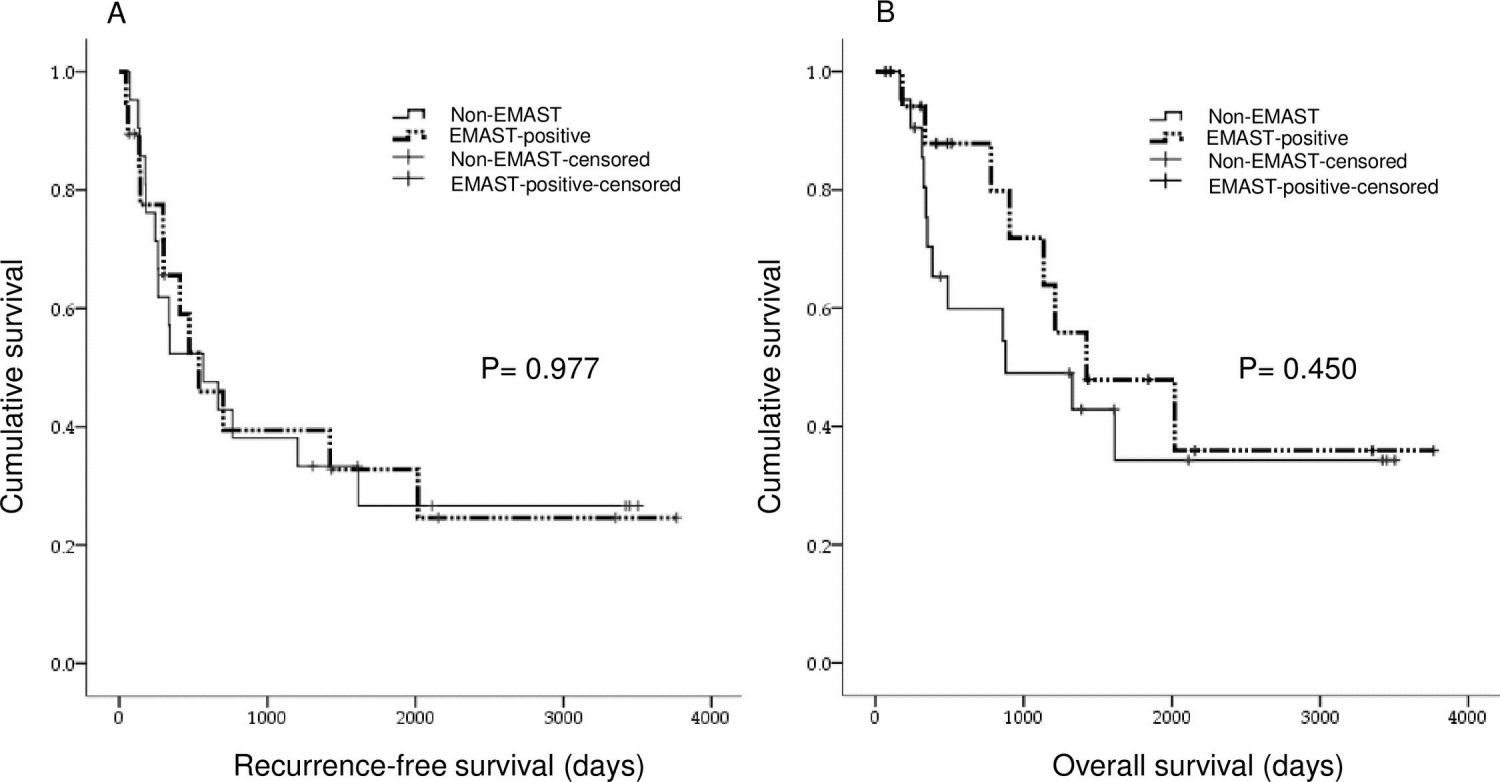
Kaplan-Meier plots of cumulative recurrence-free survival and overall survival in elevated microsatellite alterations at selected tetranucleotide repeats (EMAST)-positive tumors compared with non-EMAST tumors. (A) Cumulative recurrence-free survival of patients with EMAST-positive tumors compared with non-EMAST positive tumors (P = 0.98, log-rank test). (B) Cumulative overall survival of EMAST-positive tumors compared with non-EMAST tumors (P = 0.45, log-rank test).

We also estimated RFS and OS via Kaplan-Meier analysis (Fig 1A and 1B). Forty patients had a median OS of 1327 d (95% CI, 774–1879 d) and a median RFS of 537 d (95% CI, 162–911 d). EMAST-positive patients had a median OS of 1423 d (95% CI, 322–2523 d) and a median RFS of 537 d (95% CI, 177–896 d). Non-EMAST patients had a median OS of 878 d (95% CI, 0–1982 d) and a median RFS of 567 d (95% CI, 72–1062 d). No significant difference in OS and RFS was observed between EMAST-positive and non-EMAST patients (P = 0.45 and P = 0.98, respectively).

**Fig 2:**
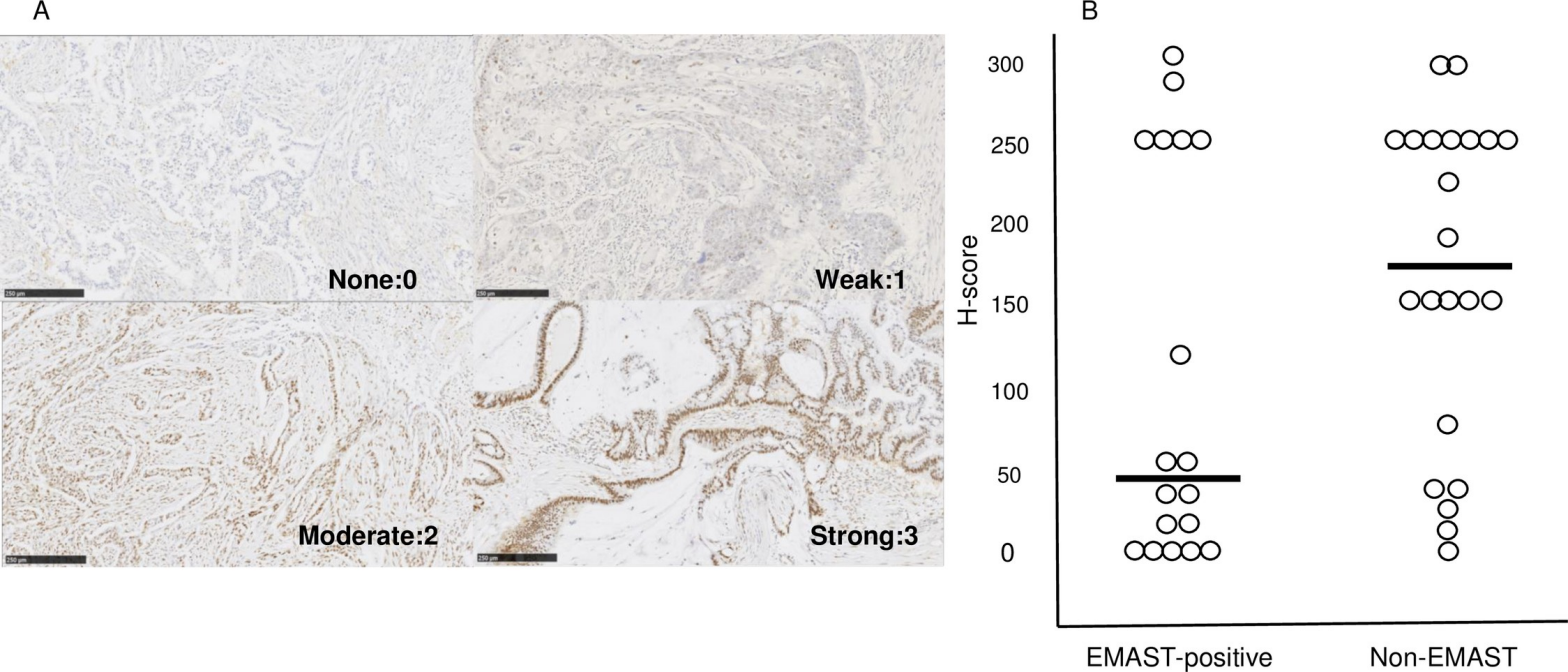
MSH3 protein expression levels in PDAC tissue specimens. (A) Histoscore (H-score) was calculated via assessment of both the percentage of positive cells and staining intensity (graded as 0, non-staining; 1, weak; 2, median; or 3, strong using adjacent normal mucosa as the median). The H-scores ranged from 0 to 300. (B) Distribution of the H-score is shown in the dot-plot graph.

IHC was performed to evaluate MSH3 protein expression levels in PDAC tissue specimens (Fig 2A). The H-score of EMAST-positive patients ranged from 0 to 300 (median, 40) and that of non-EMAST patients ranged from 0 to 300 (median, 170). MSH3 protein expression tended to be often detected in non-EMAST specimens and frequently absent or downregulated in EMAST-positive specimens. The H-score distribution is shown in Fig 2B. No significant difference in H-score was observed between EMAST-positive and non-EMAST specimens (P = 0.07).

## Discussion

This study investigated the prevalence of EMAST in PDAC and the effect of EMAST on clinicopathological factors. The present results indicate that approximately 50% of PDAC patients were EMAST-positive. This is the first report, to our knowledge, to investigate the prevalence of EMAST in PDAC.

EMAST reportedly occurs in several tumors [28–30]. In CRC patients, EMAST-positive patients display the lowest RFS with high probability of distant metastasis and an independent predictor of recurrent metastasis from stage II/III colorectal cancer [19]. Thus, the presence of EMAST is a significantly poor prognostic factor for CRC patients. In the present study, no significant difference was observed between clinicopathological factors, including OS and RFS, and EMAST status in PDAC patients.

Hence, EMAST cannot be considered a prognostic marker in PDAC. However, it can possibly serve as a biomarker for chemosensitivity because EMAST and 5-FU sensitivity are reportedly correlated in CRC [12, 25]. Since only surgical cases were considered in the present study, few cases were included. We intend to perform further verification in the future.

The reason underlying MSH3 downregulation in PDAC is not yet known. Approximately 15% of colorectal cancers exhibit MSI-H resulting from biallelic inactivation of MMR genes such as MLH1 or MSH2 [31, 32]. An (A) 8 in the coding domain of MSH3 reportedly often undergoes frameshift mutations in MSI-H tumors, resulting in loss of MSH3 protein expression [17]. Recent reports identified dMMR/MSI in only 1% of PDAC cases [20–22]. Our results are concurrent with previous results. Therefore, another mechanism, other than MSI-H, can be considered as a cause of MSH3 downregulation. *MSH3* mutations in PDAC have not been reported despite several whole-genome sequencing and exosome sequencing studies in the past [21]; hence, certain epigenetic changes may have downregulated MSH3. Recent studies reported that EMAST might be caused by loss of MSH3 in cancer cells owing to the effects of tumor micro-environmental factors, such as hypoxia and inflammation [24, 26]. The hypoxic state of tumors reduces the activity of the oxygen-dependent ten-eleven translocation (TET) enzyme group that catalyzes DNA demethylation via oxidation of 5-methylcytosine and promoter methylation [33]. As another mechanism, oxidative stress induces nucleus-to-cytoplasm translocation of MSH3 away from its functional site instead of reducing its total expression level [24].

Furthermore, mislocalization of MSH3 occurs not only with low oxygenation but also owing to inflammatory cytokines such as IL-6. Inflammatory cell infiltration, including macrophages and neutrophils, was observed in tumor specimens compared to the non-cancer portion, and inflammatory cytokines such as IL-6 were upregulated. Furthermore, mislocalization of MSH3 by inflammatory cytokines has been reported and is presumed to cause EMAST [13]. IL-6 levels are reportedly considered a prognostic marker of PDAC [34]. We investigated the association between IL-6 and EMAST via IHC in a clinical specimen. We speculated that IL-6 tended to be often expressed in non-EMAST cases; however, there was no significance difference in the H-score between EMAST-positive and non-EMAST patients, and MSH3 was not mislocalized in EMAST-positive patients.

MSH3 is reportedly involved not only in mismatch repair but also in homologous recombination, a major repair mechanism of DNA double-strand break (DSB) repair [35]. Loss of homologous recombination results in defective double-strand break repair and allows for the formation of a loss of heterozygosity (LOH) phenotype in tumors. Loss of MSH3 function results in DNA repair via non-homologous end joining (NHEJ), a repair mechanism other than the HR pathway, and LOH increases owing to low precision repair [36]. If the target gene of LOH induced by the loss of MSH3 expression is clarified, it may help elucidate the underlying mechanism and the stage at which recurrence and chemosensitivity are observed.

The quantity and quality of DNA extracted from FFPE specimens were low; hence, we could not investigate LOH in this study. Advancements in next-generation sequencing will help elucidate the association between EMAST and LOH.

NHEJ, a mechanism underlying the repair of DSB in DNA, is regulated partly by the serine/threonine kinase, a DNA dependent protein kinase (DNA-PK). The DNA-PK holoenzyme acts as a foothold protein binding disconnected DNA ends and recruiting other repair molecules. MSH3 expression levels were reported as the most significant predictors of DNA-PK inhibition [35]. DNA-PK inhibition resulted in apoptosis in MSH3 mutant cell lines *in vitro* and showed considerable efficacy against MSH3 mutant tumors *in vivo*. Recently, specific DNA-PK inhibitors displayed efficient synergistic effects of chemotherapy *in vitro* [35]. DNA-PK inhibition is expected to be a therapeutic alternative to treat human cancers presenting defects in homologous recombination. We are currently investigating the sensitivity of DNA-PK inhibitors *in vitro*.

Previously, IHC-based MSI testing for MLH1 and MSH2 provided a sensitive (92.3%) and an extremely specific (100%) method for screening defects in DNA mismatch repair in CRC patients [37]. Consequently, IHC-based MSI testing is reportedly highly concordant with DNA-based MSI analysis. The correlation between protein expression levels of MSH3 via IHC and PCR-based EMAST analysis had not been conducted in cases of PDAC. In the present study, no significant positive associations were observed between EMAST status and MSH3 protein expression levels upon IHC; however, with subsequently large number of cases, a significant difference may be observed. If EMAST status can be evaluated via IHC, EMAST may be evaluated using a small amount of specimen via methods such as endoscopic ultrasound fine-needle aspiration (EUS-FNA).

In conclusion, the present retrospective analysis indicates that approximately 50% of PDAC patients were EMAST-positive. This is the first study, to our knowledge, to examine the prevalence of EMAST in PDAC patients. Although no significant difference was observed between the clinicopathological factors including OS and RFS with EMAST status in PDAC, further studies are required to elucidate the role of EMAST status in disease progression and chemosensitivity.

## Supporting information

S1 Table Primer sequence for five polymorphic tetranucleotide markers and five mononucleotide markers. (DOCX)
