# Supplementary material for: Prevalence of elevated microsatellite alterations at selected tetranucleotide repeats in pancreatic ductal adenocarcinoma
Source: PLoS One. 2018 Dec 7;13(12):e0208557. doi: 10.1371/journal.pone.0208557 (PMC6285458; doi:10.1371/journal.pone.0208557)
Supplement: S1 Table — (DOCX) [file pone.0208557.s001.docx]

**S1 Table.** Primer sequence for five polymorphic tetranucleotide markers and five mononucleotide markers

| Primer | Primer sequence | 5' modification |
| --- | --- | --- |
| MYCL1 | Forward: 5'-TGGCGAGACTCCATCAAAG-3' | 6-FAM |
|  | Reverse: 5'-CCTTTTAAGCTGCAACAATTTC-3' |  |
| D9S242 | Forward: 5'-GTGAGAGTTCCTTCTGGC-3' |  |
|  | Reverse: 5'-ACTCCAGTACAAGACTCTG-3' | 6-FAM |
| D20S82 | Forward: 5'-GCCTTGATCACACCACTACA-3' |  |
|  | Reverse: 5'-TGTGGTCACTAAAGTTTCTGCT-3' | 6-FAM |
| D20S85 | Forward: 5'-GAGTATCCAGAGAGCTATTA-3' |  |
|  | Reverse: 5'-ATTACAGTGTGAGACCCTG-3' | 6-FAM |
| D8S32 | Forward: 5'-GATGAAAGAATGATAGATTACAG-3' |  |
|  | Reverse: 5'-ATCTTCTCATGCCATATCTGC-3' | 6-FAM |
| BAT-25 | Forward: 5'-TCGCCTCCAAGAATGTAAGT-3' | NED |
|  | Reverse: 5'-TCTGCATTTTAACTATGGCTC-3' |  |
| BAT-26 | Forward: 5'-TGACTACTTTTGACTTCAGCC-3' | 6-FAM |
|  | Reverse: 5'-AACCATTCAACATTTTTAACCC-3' |  |
| NR-21 | Forward: 5'-TAAATGTATGTCTCCCCTGG-3' | VIC |
|  | Reverse: 5'-ATTCCTACTCCGCATTCACA-3' |  |
| NR-22 | Forward: 5'-GAGGCTTGTCAAGGACATAA-3' |  |
|  | Reverse: 5'-AATTCGGATGCCATCCAGTT-3' | 6-FAM |
| NR-24 | Forward: 5'-CCATTGCTGAATTTTACCTC-3' |  |
|  | Reverse: 5'-ATTGTGCCATTGCATTCCAA-3' | VIC |
